# Comparative safety and efficacy of pharmacological and non-pharmacological interventions for the behavioral and psychological symptoms of dementia: protocol for a systematic review and network meta-analysis

**DOI:** 10.1186/s13643-017-0572-x

**Published:** 2017-09-07

**Authors:** Jennifer Watt, Zahra Goodarzi, Andrea C. Tricco, Areti-Angeliki Veroniki, Sharon E. Straus

**Affiliations:** 1 Department of Geriatric Medicine, University of Toronto, 27 King’s College Circle, Toronto, ON M5S 1A1 Canada; 2 Institute for Health Policy Management and Evaluation, University of Toronto, 4th Floor, 155 College Street, Toronto, ON M5T 3M6 Canada; 3 Section of Geriatric Medicine, Department of Medicine, University of Calgary, Calgary, Alberta Canada; 4 Li Ka Shing Knowledge Institute, St. Michael’s Hospital, 209 Victoria Street, East Building, Room 716, Toronto, ON M5B 1W8 Canada; 5 Epidemiology Division, Dalla Lana School of Public Health, University of Toronto, Health Sciences Building, 155 College Street, 6th floor, Toronto, ON M5T 3M7 Canada

**Keywords:** Behavioral and psychological symptoms of dementia (BPSD), Pharmacological treatments, Non-pharmacological treatments, Dementia, Network meta-analysis, Systematic review, Knowledge synthesis

## Abstract

**Background:**

Behavioral and psychological symptoms of dementia (BPSD) are highly prevalent in patients with dementia. Both pharmacological and non-pharmacological strategies are commonly used to treat these symptoms, but their comparative safety and efficacy is unknown.

**Methods:**

We will conduct a systematic review of the published and unpublished literature to retrieve all articles pertaining to outcomes of safety and efficacy associated with pharmacological and non-pharmacological treatments of BPSD for patients living in the community and institutionalized care settings. Our primary outcome of efficacy is a change in aggression. Our primary outcome of safety will be risk of fracture. These primary outcomes were chosen by stakeholders involved in the care of patients experiencing BPSD. Possible secondary outcomes of efficacy will include a change in agitation, depressive symptoms, and night-time behaviors. Possible secondary outcomes of safety will include the risk of stroke, falls, and mortality. All article screening, data abstraction, and risk of bias appraisal will be completed independently by two reviewers. If the assumption of transitivity is valid and the evidence forms a connected network, Bayesian random-effects pairwise and network meta-analyses (NMAs) will be conducted. Relative treatment rankings will be reported with mean ranks and the surface under the cumulative ranking curve.

**Discussion:**

We will identify the safest and most efficacious treatment strategies for patients with BPSD from among our most highly ranked treatments. The results of this study will be used to guide decision-making and improve patient care.

**Systematic review registration:**

PROSPERO registry number CRD42017050130.

**Electronic supplementary material:**

The online version of this article (10.1186/s13643-017-0572-x) contains supplementary material, which is available to authorized users.

## Background

Dementia is a progressive neurodegenerative disorder characterized by both cognitive and functional impairment [1]. In 2010, it was estimated that 35.6 million people were living with dementia worldwide and this number was projected to double over the subsequent 20 years [2]. Importantly, 75% of people with dementia manifest behavioral and psychological symptoms of dementia (BPSD) in a given month (e.g., aggression, agitation, and apathy), which can lead to significant distress for caregivers [3–5].

Numerous pharmacological and non-pharmacological treatments have been proposed that target BPSD symptoms [6]. Exercise, animal therapy, and reminiscence therapy are non-pharmacological approaches that are possible interventions to improve the symptoms of BPSD [6, 7]. Commonly used pharmacological approaches for BPSD include antipsychotics, antidepressants, and cholinesterase inhibitors [8, 9]. In a study by Kales et al., it was found that 28.8% of patients with dementia were prescribed antipsychotics; however, these medications are associated with several serious adverse events including fractures, pneumonia, stroke, myocardial infarction, and acute kidney injury [10–15]. Except in the case of a patient endangering self or others, many guidelines support the use of non-pharmacological strategies prior to initiating a pharmacological approach to symptom management [16]. This recommendation is not consistent across treatment guidelines, which may relate to the lack of head-to-head studies examining the comparative efficacy and safety of these different interventions [9].

Given the concerns about drug safety in patients with BPSD, it is critical to understand the comparative safety and efficacy of pharmacological and non-pharmacological interventions. To our knowledge, a network meta-analysis describing the comparative safety and efficacy of these two types of treatment strategies has not been previously completed. As such, our objective is to conduct a systematic review and network meta-analysis on this topic.

## Methods

This protocol is written in accordance with the Preferred Reporting Items for Systematic Review and Meta-Analysis Protocols (PRISMA-P) (see Additional file 1. PRISMA Checklist) [17]. Any amendments to this protocol will be reflected in an update to the PROSPERO registration. The final publication of study findings will be written in accordance with the PRISMA extension for network meta-analyses and the International Society for Pharmacoeconomics and Outcomes Research (ISPOR) Indirect Treatment Comparison/Network Meta-Analysis Study Questionnaire to Assess Relevance and Credibility to Inform Health Care Decision-Making [18, 19].

## Eligibility criteria

### Population

Our study population will include all patients with a diagnosis of dementia, as defined by study authors (e.g., medical history of dementia, diagnostic and statistical manual (DSM) diagnosis of major neurocognitive disorder), residing in the community or an institutionalized setting. There will be no restrictions based on patient age, severity of dementia, or type of dementia [1].

### Interventions

Any pharmacological or non-pharmacological treatment strategy for the behavioral and psychological symptoms of dementia (BPSD) will be considered for study inclusion in our analyses for efficacy outcomes; however, pharmacological treatments must have received final approval by the Federal Drug Agency or Health Canada, as of the date of our literature search. Given that some treatments for BPSD (e.g., antipsychotics) are used in an “off-label” fashion, we do not want to exclude potential interventions from our analyses of efficacy based on their approved indications. We will limit our analyses of safety to the following pharmacological interventions: antipsychotics, antidepressants, sedative/hypnotics, mood stabilizers, anticonvulsants, stimulants, cholinesterase inhibitors, and N-methyl-D-aspartate (NMDA) receptor antagonists. We limited our systematic review of safety outcomes for two reasons: (1) safety outcomes were not consistently reported in studies of non-pharmacological interventions in our preliminary searches, and (2) many non-pharmacological interventions, in particular, are used to treat conditions other than BPSD in our patient population, which would have made our systematic review unfeasible to carry out in a timely manner. Examples of potential pharmacological and non-pharmacological interventions are outlined in Table 1 [6].

**Table 1:** Examples of pharmacological and non-pharmacological interventions for behavioral and psychological symptoms of dementia (BPSD)

*Pharmacological interventions*	
Antipsychotics (e.g., risperidone)	
Antidepressants (e.g., citalopram)	
Cholinesterase inhibitors (e.g., donepezil)	
Memantine	
Benzodiazepines (e.g., lorazepam)	
Anticonvulsants (e.g., valproate)	
Mood stabilizers (e.g., lithium)	
Stimulants (e.g., methylphenidate)	
Others (e.g., melatonin, quinidine)	
*Non-pharmacological interventions*	
Reminiscence therapy	
Validation therapy	
Simulated presence therapy	
Light therapy	
Animal therapy	
Aromatherapy	
Snoezelen room	
Exercise	
Cognitive training and rehabilitation	

### Comparators

Eligible comparator groups within studies will include usual care or another pharmacological or non-pharmacological treatment strategy for BPSD.

### Outcomes

Our primary outcomes were chosen by a convenience sample of 12 stakeholders from Ontario and Alberta with diverse experience in caring for patients with BPSD (e.g., nurses, geriatricians, caregivers of patients with dementia, and allied health professionals). Based on the ranked preferences of these stakeholders, the primary outcome of treatment efficacy will be patient aggression. Other possible secondary outcomes of treatment efficacy are outlined in Table 2 (e.g., depressive symptoms, neuropsychiatric inventory total score). The primary outcome of treatment safety will be the risk of fracture. The risk of fracture will be measured as a dichotomous outcome (fracture versus no fracture). Overall estimates of mortality will also be presented as secondary outcomes. Other possible secondary outcomes of treatment safety are outlined in Table 2 (e.g., falls, stroke).

**Table 2:** Examples of outcomes associated with treatment of behavioral and psychological symptoms of dementia (BPSD)

*Outcomes of treatment efficacy*	
Aggression*	
Agitation	
Depression	
Apathy	
Anxiety	
Night-time behaviors	
Motor disturbances	
Irritability	
Disinhibition	
Psychosis (delusions and hallucinations)	
Appetite and eating abnormalities	
Caregiver well-being	
Overall quality of life (both patient and caregiver)	
Improvement in function	
Admission to skilled nursing facility	
*Outcomes of treatment safety*	
Fractures*	
Falls	
Stroke	
Mortality	
Prolonged QTc interval	
Myocardial infarction	
Pneumonia	
Deterioration in cognition	
Hyperprolactinemia	
Weight gain	
Somnolence	
Hypotension	
Edema	
Cytopenia	
Extrapyramidal side effects (e.g., tardive dyskinesia)	
Hospitalization	
Admission to intensive care unit (ICU)	

*Primary outcome of interest

### Study designs

Only randomized controlled trials (RCTs) will be included in our systematic review of efficacy outcomes. The following study designs will be eligible for inclusion in our systematic review of safety outcomes: RCTs, quasi-randomized controlled trials, non-randomized controlled trials, controlled-before-and-after studies, interrupted time series, cohort studies, and case-control studies. We plan to include observational studies in our systematic review of safety outcomes because of their importance in identifying adverse drug events in the BPSD literature [20, 21]. Case series, case reports, and qualitative studies will be excluded. Systematic reviews related to the topic will be retained to search their references for potential, eligible studies.

## Information sources and search strategy

An information specialist developed a search strategy for our clinical question (see Additional file 2. MEDLINE search strategy), which was peer-reviewed by a second information specialist using the Peer Review of Electronic Search Strategies (PRESS) checklist [22]. The following databases will be searched for citations published in any language: MEDLINE, EMBASE, the Cochrane Library, CINAHL, and PsycINFO. Searches of the difficult to locate/unpublished (or gray) literature will be conducted using websites (e.g., Government of Canada website), search engines (e.g., Google Scholar, the Turning Research into Practice (TRIP) database), and thesis databases (e.g., Center for Research Libraries Foreign Dissertation). Reference lists of included studies and related systematic reviews will be scanned to identify additional studies for inclusion in our systematic review. Content experts from Toronto and Calgary who ranked the outcomes will be contacted via email for additional relevant studies.

## Data collection and analysis

### Study selection

Two levels of screening will be completed independently using *Synthesi*.SR (proprietary online software developed by the Knowledge Translation Program of St Michael’s Hospital, Toronto, Canada, http://www.breakthroughkt.ca/login.php). Two reviewers will independently review the title and abstract of articles retrieved from the literature search to determine if a study is eligible for inclusion. At the initiation of article screening, a calibration exercise will occur whereby each reviewer will independently screen 10% of a random sample of articles to ensure appropriate inter-rater agreement (at least 80% agreement). Discrepancies between the two reviewers will be resolved by consensus; otherwise, a third reviewer will be available to make a final decision about an article’s inclusion. The full-text of articles retained from level one screening will then be reviewed to confirm each article’s eligibility for inclusion. If a conference abstract is retained for level two screening, study authors will be contacted for further information as to whether a related manuscript has subsequently been published or to ensure the study meets our outlined eligibility criteria, as required. Whenever it is unclear if a study meets our outlined eligibility criteria, authors will be contacted for further information.

### Data abstraction

Prior to data abstraction, we will complete a charting exercise to better inform the structure of our data abstraction form in terms of: (1) types of studies retrieved, (2) outcomes reported, and (3) effect measures used by study authors [23]. All data will be abstracted independently by two reviewers from those studies retained in level two screening using a data abstraction form. The form will be piloted by each team member on a random sample of five included studies to ensure adequate inter-rater reliability (at least 80% agreement). The form will be modified as necessary to ensure clarity for reviewers based on our charting exercise. Disagreements will be resolved by a third person. When multiple studies report data from the same study population (e.g., companion reports), the study with the primary outcome of interest (fractures or aggression) or the largest sample size will be considered the major publication and the others will be retained for supplementary material only.

Information to be abstracted as potential effect modifiers will include study characteristics (e.g., year of study publication, authorship, location(s) of study, journal of publication, study sponsorship), patient characteristics (e.g., average (mean or median) age of study population, proportion of female patients, care setting, type(s) of dementia, severity of dementia, and standard of care in each care setting), and intervention characteristics (e.g., to whom the intervention was directed (e.g., patient, caregiver, clinician, and surrounding environment), and details of the intervention (e.g., intervention protocol or medication dosing schedule)).

Primary and secondary arm- or trial-level outcomes associated with intervention safety and efficacy (Table 2) will be extracted from included studies. Outcomes of efficacy and safety will be extracted at short-term (≤30 days), medium-term (31–364 days), and long-term (>364 days) follow-up because many interventions have been evaluated at many different time-points in our preliminary searches [7, 24, 25]. All doses and schedules of drug administration will be extracted from included studies.

### Node formation

We expect this review to identify numerous interventions for BPSD. There is no established taxonomy for classifying interventions for BPSD; however, we will begin with the broad categories of patient-, care provider-, and environment-oriented interventions [6]. In order to build a framework, we propose a qualitative consensus-based categorization procedure [26]. This will involve the following four steps by two researchers at each step: (1) identifying, coding and defining all interventions from the systematic review, (2) independent categorization of interventions into relevant domains (e.g., all interventions coded as relating to a multi-sensory intervention would be sorted into this domain), (3) resolving any discrepancies in the categorization of interventions through discussion, and (4) emailing a representative group of stakeholders (e.g., clinicians, caregivers, allied health professionals) to review, reach consensus through discussion, and finalize the domains. This will provide feedback and ensure stakeholder validation of the proposed domains. At the initiation of step one, a calibration exercise will occur whereby each reviewer will independently identify, code, and define interventions from 10% of a random sample of articles to ensure appropriate inter-rater agreement (at least 80% agreement). This process ensures a rigorous approach to the categorization of interventions by using a qualitative method of independent multiple coding of the interventions and a consensus approach integrating the stakeholders early in the analysis.

### Risk of bias and quality assessment

Risk of bias assessment of each included study will be completed independently by two reviewers. If there is disagreement between reviewers, a third reviewer will be available. In the case multiple outcomes are reported in a single study, we will use the hierarchy outlined by Kirkham et al., to establish our order of preference for selecting an outcome on which to complete our assessment of bias [27].

The risk of bias of included clinical trials will be assessed as per the methodology of the Cochrane Handbook for Systematic Reviews of Interventions [28]. The quality assessment of observational studies will be assessed with the Newcastle-Ottawa scale [29]. In the assessment of case-control and cohort studies, a control patient might also be living in an institutional setting, in which case the study would still be awarded a star for an appropriate selection of control group. The most important confounder to adjust for in an observational study would be age, but other important confounders will include sex, comorbidities, dementia severity, caregiver availability, care setting, and other current or prior treatments for BPSD. For certain outcomes of intervention efficacy (e.g., change in aggression or agitation), the symptom may be present at the start of the study, but still be awarded a star if a change from baseline is reported. An appropriate length of follow-up for safety outcomes could be as little as 30 days, while most studies of efficacy outcomes would be expected to last at least 4 to 6 weeks—many are 10 weeks or longer [24, 30]. We plan to assess other study designs with the Cochrane Effective Practice and Organization Care Risk-of-Bias Tool [31].

### Measures of treatment effect

If studies consistently report continuous data outcomes that are measured on the same scale, then mean differences (MDs) will be used. An odds ratio (OR) will be used if studies report an outcome as dichotomous data. To derive summary effect measures that combine both dichotomous and continuous effect measures, MDs or standardized mean differences (SMDs) will be transformed to OR estimates [28, 32, 33]. For outcomes that are reported with a number of different scales across studies, the SMD will be derived and will be transformed into an OR to facilitate the outcome’s interpretation by knowledge users [34]. The order of preference for selecting source data, when multiple options are reported by study authors (e.g., 2 × 2 tables, adjusted and unadjusted ORs, MDs, SMDs) is described in Additional file 3 (Additional file 3. Order Preference for Combining Data Types). In the case where authors report several scales for the same outcome, we will use our charting exercise to better inform our choice of scale used in the derivation of our summary effect measures. If a cluster design is reported, outcome measures will be extracted from the primary study that account for the clustering; however, if these data are not available, then the method of Rao and Scott will be used to account for the correlation in these data [28, 35, 36]. For the presentation of results, the summary relative effect sizes (e.g., MDs or ORs) and associated 95% credible intervals (CrIs) for each possible pairwise comparison will be used.

### Missing data

Where adjusted summary effect measures are reported, study-level data as provided by study authors will be included in our analyses. The type of data imputation method used for missing data will be noted on our data abstraction form so that the quality assessment of each study will reflect the appropriateness of the data imputation method used to account for missing data. For example, attrition in a trial for dementia treatments may be related to side effects of the treatment, and using the last observation carried forward approach can introduce important bias favoring the treatment, as outcomes tend to deteriorate with time. Informative missing odds ratios (IMORs) for dichotomous outcomes and informative missingness difference of means (IMDoM) for continuous outcomes will be derived to capture the uncertainty in our estimates from missing data under the missing at random assumption [37, 38]. For continuous outcomes that are not reported as means with associated standard deviations, imputation methods will be used to derive approximate effect measures [28, 39]. Study authors will be contacted for further information prior to applying data imputation methods, as needed.

### Assessment of transitivity

The assumption of transitivity will be assessed to ensure that potential effect modifiers described above are balanced on average across treatment comparisons [40, 41]. Treatment groups receiving the standard of care (or placebo) will be evaluated to ensure they are similar across pairwise comparisons [42].

## Data synthesis

Included studies will be summarized descriptively based on study characteristics, study-level patient covariates, interventions and outcomes studied, and our assessment of risk of bias. If quantitative synthesis is not appropriate, we will narratively describe the findings of our systematic review. In our pairwise and network meta-analyses of treatment efficacy and safety, we will pool effect measures across all types of dementia because studies in the BPSD literature frequently include patients with any type of dementia, to derive overall effect estimates based on the totality of the evidence; however, in our secondary analysis of treatment efficacy we will not assume exchangeability of priors for between-study heterogeneity across dementia subtypes given their well-described differences in presentation and clinical course [1, 30, 43]. We will further test our findings in subgroup analyses based on dementia subtype.

### Direct treatment comparisons

Bayesian random-effects models using vague priors for all trial baselines (*N*(0,1000)), treatment effects (*N*(0,1000)), and between-study standard deviations (σ ~ *N*(0,100) for *σ* >0) will be used to derive summary effect measures with associated 95% credible intervals when two or more studies report data that can be included in the analysis [44].

### Indirect and mixed treatment comparisons

Outcomes of treatment efficacy will be modeled as described in Dias et al., if the assumption of transitivity is valid and the evidence forms a connected network [45, 43]. A three-level hierarchical model as described in Schmitz et al., will be used to model outcomes of treatment safety given that we will be including both randomized and non-randomized study designs [43]. Random-effects models are most appropriate given the anticipated clinical and methodological heterogeneity among pooled studies [28]. We will assume vague prior distributions for all trial baselines (*N*(0,1000)), treatment effects (*N*(0,1000)), and between-study standard deviations (*σ* ~ *N*(0,100) for *σ* >0). We will use a minimally informative prior for between-study type standard deviations (*N*(0,1) for *γ* >0), which is consistent with priors used in previous Bayesian 3-level hierarchical NMA models [21, 43].

Model convergence will be assessed using the Brooks-Gelman-Rubin diagnostic and goodness of model fit will be assessed with the deviance information criterion [46]. These analyses will be completed using JAGS software [47]. Relative treatment rankings will be reported with mean ranks and the surface under the cumulative ranking curve [48]. We will present tables in our final manuscript that contain the rank probabilities of each intervention and associated efficacy and safety outcomes given the uncertainty related to the interpretation of intervention rankings [49]. Number needed to treat for an additional beneficial outcome (NNTB) and number needed to treat for an additional harmful outcome (NNTH) will be estimated for each intervention [28, 50]. Rank-heat plots will be used to display the treatment rankings across multiple outcomes [51].

### Assessment of inconsistency

Global consistency of the entire network will be assessed with the design-by-treatment interaction model [52]. If inconsistency is found within the network, local inconsistency of the loops within each network will then be assessed with the loop-specific approach to generate an inconsistency factor with an associated 95% CI [53–55].

### Exploring sources of heterogeneity or inconsistency with subgroup analyses and meta-regression

Subgroup analyses will be undertaken to explore the influence of potential effect modifiers further. If there are a sufficient number of studies identified reporting study-level data to assess our hypothesized effect modifiers, we will perform analyses based on subgroups of the following effect modifiers: age, sex, severity of dementia, dementia type, care setting, availability of caregiver, specialty of treating clinician, and number of prior treatments trialed. Network meta-regression will be used to explore the effect of study year if more than 10 studies are available.

### Sensitivity analyses

The robustness of our study findings will be tested with the following sensitivity analyses (in addition to the aforementioned sensitivity analyses) incorporating only data from the following studies into the network estimates: (1) RCTs (outcomes of safety only), (2) RCTs and cohort studies reporting effect measures that are adjusted for important confounders (outcomes of safety only), (3) studies at low risk of bias based on the two components of our risk of bias assessment found to be the greatest threat to study validity [4], studies at low or moderate risk of bias based on the two components of our risk of bias assessment found to be the greatest threat to study validity, and [5] studies where study authors use a standardized method for the diagnosis of dementia. Our choice of priors on the between-study standard deviation will be tested in sensitivity analyses with the following vague priors: *σ* ~ *U*(0,10) and log(*σ*) ~ *N*(0,1000).

### Assessment of publication bias and small-study effects

We will use contour-enhanced funnel plots for each treatment comparison separately to assess for publication bias if there are 10 or more studies reporting on a particular outcome [28, 56]. Within each funnel plot, we will distinguish cohort studies from RCTs and we will also illustrate study quality by using distinct symbols. Small-study effects will be tested within a network meta-regression model that distinguishes studies based on their size [57].

## Dissemination of study findings

Early stakeholder involvement improves knowledge dissemination, as stakeholders are engaged from question formation to the end of study activities [58]. Given the complexity of BPSD management and the need to improve care at the bedside, the early involvement of stakeholders will potentially improve the impact of this research.

As such, we aim to use an integrated knowledge translation approach, with early participation and engagement of knowledge/end users in the research process in the following ways: (1) surveying of knowledge/end users to identify their preferences for primary efficacy and safety outcomes, (2) integrating a qualitative consensus-based categorization procedure (as described in *Node Formation*) into our verification process for ensuring the proper categorization of interventions, and (3) discussing study findings with stakeholder groups to understand the broader social context of our findings given the importance of social constructs such as gender in the caregiving role of patients with BPSD and to identify key messages for study findings [26, 59, 60].

For dissemination, we will pursue open access publication(s) and presentation of results at several local, national, and international meetings. Local dissemination will take place in two provinces (Ontario and Alberta). We will engage with patient advocacy groups through our stakeholder linkages to disseminate our results through their media platforms and create a research brief to post on the St. Michael’s Hospital Knowledge Translation Program website.

## Discussion

There will be two main anticipated challenges in completing this systematic review and network meta-analysis: (1) incorporating both randomized and non-randomized study designs into our network meta-analyses for safety outcomes, and (2) ensuring the treatment comparisons in our networks maintain transitivity in our network meta-analyses while also remaining clinically meaningful to knowledge users. The decision to incorporate both randomized and non-randomized study designs into the network meta-analyses for safety outcomes will provide knowledge users (e.g., clinicians, caregivers) with a better understanding of the risks associated with possible treatment strategies given the significant number of publications in the observational literature on this topic; however, there are methodological concerns about the possibility of unknown confounders that could affect the validity of our findings [44, 61]. It is also important to further explore the findings of industry-sponsored studies in a real-world setting to capture adverse drug events that RCTs are not powered to detect, which is often done with observational study designs [62].

Optimal organization of our treatment nodes within our networks will also be a challenge. Integrating non-pharmacological interventions into our network meta-analysis will require input from both researchers and knowledge users, which is why we will utilize a qualitative consensus-based categorization procedure [26]. Complex non-pharmacological interventions have previously been integrated into network meta-analyses [63, 64]. We hope that by including non-pharmacological interventions into our network meta-analyses of efficacy outcomes that we can help knowledge users to make informed health care decisions concerning the management of BPSD.

## Additional files

Additional file 1: PRISMA Checklist. (DOCX 36 kb)

Additional file 2: MEDLINE Search Strategy. (DOCX 16 kb)

Additional file 3: Order Preference for Combining Data Types. (DOCX 14 kb)

## Abbreviations

BPSD: Behavioral and psychological symptoms of dementia
CIHR: Canadian Institutes for Health Research
IMDoM: Informative missingness difference in means
IMOR: Informative missing odds ratios
ISPOR: International Society for Pharmacoeconomics and Outcomes Research
MD: Mean difference
NMA: Network meta-analysis
NMDA: N-methyl-D-aspartate
NNTB: Number needed to treat
NNTH: Number needed to harm
OR: Odds ratio
PRESS: Peer Review of Electronic Search Strategies
PRISMA-P: Preferred reporting items for systematic review and meta-analysis protocols
RCT: Randomized controlled trial
SMD: Standardized mean difference
TRIP: Turning Research into Practice
