# Supplementary material for: Comparative safety and efficacy of pharmacological and non-pharmacological interventions for the behavioral and psychological symptoms of dementia: protocol for a systematic review and network meta-analysis
Source: Syst Rev. 2017 Sep 7;6:182. doi: 10.1186/s13643-017-0572-x (PMC5590133; doi:10.1186/s13643-017-0572-x)
Supplement: Supplementary file 2 — MEDLINE Search Strategy. (DOCX 16 kb) [file 13643_2017_572_MOESM2_ESM.docx]

# Additional File 2. MEDLINE Search Strategy

1    exp dementia/ (154751)
 2    exp Parkinsonian Disorders/ (72636)
 3    (dementia* or amentia* or Alzheimer* or Creutzfeldt-Jakob Syndrome* or Primary Progressive aphasia or Primary Progressive Nonfluent Aphasia* or Primary Progressive Non-fluent Aphasia* or Frontotemporal Lobar Degeneration* or Fronto-temporal Lobar Degeneration* or CADASIL or Huntington* or Kluver-Bucy Syndrome* or Lewy Bod*).tw. (199793)
 4    (Parkinson* or paralysis agitans).tw. (98705)
 5    (Pick* disease* or lobar atroph* or kosaka shibayama disease* or neurofibrillary tangle* or neuro-fibrillary tangle*).tw. (10499)
 6    or/1-5 (314937)
 7    exp Stress, Psychological/ (116719)
 8    mental disorders/ (153511)
 9    exp anxiety disorders/ (76029)
10    exp "disruptive, impulse control, and conduct disorders"/ (8398)
11    exp dissociative disorders/ (4024)
12    exp elimination disorders/ (5281)
13    exp "feeding and eating disorders"/ (27618)
14    exp mood disorders/ (114867)
15    exp neurotic disorders/ (18536)
16    exp sleep wake disorders/ (77814)
17    exp somatoform disorders/ (18519)
18    exp psychotic disorders/ (50005)
19    affective disorders, psychotic/ or capgras syndrome/ or delusional parasitosis/ or morgellons disease/ or paranoid disorders/ (6920)
20    (Affective Disorder* or Capgras Syndrome* or Delusional Parasitos#s or Paranoid Disorder* or Psychotic Disorder* or Sleep Wake Disorder* or Dyssomnias or Parasomnias or Somatoform Disorder*).tw. (25511)
21    (Conversion Disorder* or Factitious Disorder* or Hypochondrias#s or Neurasthen*).tw. (3428)
22    (Anxiety or anxieties or Elimination Disorder* or Encopresis or Enuresis or feeding disorder* or Eating Disorder* or Anorexia* or Binge-Eating Disorder* or Bulimia or pica or Mood Disorder* or Depressive Disorder* or depression or Cyclothymic Disorder* or Motor Disorder* or Consciousness Disorder* or delirium or Dyslexia*).tw. (443345)
 23    (Adjustment Disorder* or Stress Disorder*).tw. (24875)
 24    (psychologic* symptom* or neuro-psychiatric symptom* or neuropsychiatric symptom* or psycho-behavioral symptom* or psychobehavioral symptom* or psycho-behavioural Symptom* or psychobehavioural Symptom* or psychiatric symptom*).tw. (18418)
25    (Behavioral symptom* or behavioural symptom* or disruptive behavior* or disruptive behavior* or noncognitive symptom* or non-cognitive symptom* or neuropsychological symptom* or neuro-psychological symptom* or bpsd).tw. (7578)
26    exp *"behavior and behavior mechanisms"/ (1663125)
27    (Adaptation or Mental Competenc* or Motivation or Neurobehavioral Manifestation* or Temperance).tw. (186325)
28    Sexual Behavior/ (53374)
 29    (Sexual activit* or Sexual behavior or sexual behavior or Sexual behaviour or sexual behaviour).tw. (28260)
30    or/7-29 (2393320)
31    6 and 30 (58344)
32    randomized controlled trial.pt<[http://trial.pt<http://trial.pt/]http://trial.pt<http://trial.pt/>>. (469524) 33    controlled clinical trial.pt<[http://trial.pt<http://trial.pt/]http://trial.pt<http://trial.pt/>>. (95062) 34    (randomized or placebo or randomly).ab. (731266)
35    clinical trials as topic.sh. (189460)
36    trial.ti. (174869)
37    controlled before.tw<[http://before.tw<http://before.tw/]http://before.tw<http://before.tw/>>. (1072)
38    Controlled Before-After Studies/ (204)
39    CBA stud*.tw. (78)
40    Historically Controlled Study/ (87)
41    Interrupted Time Series Analysis/ (261)
42    ITS stud*.tw. (1423)
43    (time adj series).tw. (22456)
44    or/32-43 (1158516)
45    31 and 44 (4807)
46    animals/ not humans/ (4635010)
47    45 not 46 (4717)
48    47 not (letter not randomized controlled trial).pt. (4690)
49    47 not (comment or editorial).pt. (4677)
50    48 or 49 (4704)
51    limit 50 to english (4439)
52    Non-Randomized Controlled Trials as Topic/ (103)
53    ((nonrandom* or non-random* or quasi-random* or quasi-experiment*) adj (stud* or trial*)).tw. (11031)
54    (non-RCT or non-RCTs or nRCT or nRCTs).tw. (539)
55    exp cohort studies/ (1712991)
56    (cohort stud* or Follow-Up Stud* or Longitudinal Stud* or Prospective Stud* or Retrospective Stud*).tw. (491488)
57    exp case-control studies/ (876201)
58    case-control* stud*.tw. (90504)
59    or/52-58 (2079423)
60    31 and 59 (9436)
61    (ae or co or de or ci).fs. (5909628)
62    (safe or safety or side effect* or undesirable effect* or treatment emergent or tolerability or toxicity or adrs or (adverse adj2 (effect or effects or reaction or reactions or event or events or outcome or outcomes))).ti,ab. (1274405)
63    61 or 62 (6608728)
64    60 and 63 (3761)
65    animals/ not humans/ (4635010)
66    64 not 65 (3745)
67    66 not (comment or editorial).pt. (3727)
68    66 not (letter not randomized controlled trial).pt. (3696)
69    67 or 68 (3734)
70    limit 69 to english (3556)
71    ae.fs. (1589031)
72    62 or 71 (2528369)
73    60 and (72 or 62) (1322)
74    73 not 65 (1317)
75    74 not (comment or editorial).pt. (1308)
76    74 not (letter not randomized controlled trial).pt. (1308)
77    75 or 76 (1313)
78    limit 77 to english (1229)
