# Supplementary material for: Comparative safety and efficacy of pharmacological and non-pharmacological interventions for the behavioral and psychological symptoms of dementia: protocol for a systematic review and network meta-analysis
Source: Syst Rev. 2017 Sep 7;6:182. doi: 10.1186/s13643-017-0572-x (PMC5590133; doi:10.1186/s13643-017-0572-x)
Supplement: Supplementary file 3 — Order Preference for Combining Data Types. (DOCX 14 kb) [file 13643_2017_572_MOESM3_ESM.docx]

# Additional File 3. Order Preference for Combining Data Types

| **Type of Data** | **Pooling Preference Across Dichotomized Data** | **Pooling Preference Across All Data Types** |
| --- | --- | --- |
| Raw Data | 2 | 3 |
| Unadjusted Effect Measures | 3 | 4 |
| Adjusted Effect Measures | 1 | 1 |
| Mean Difference | Not applicable | 2 |
| Standardized Mean Difference | Not applicable | 2 |
